# Deferring Nail Mycological Sampling during the COVID-19 Pandemic: Recommendations from a Multidisciplinary Panel of Nail Specialists

**DOI:** 10.1159/000520628

**Published:** 2021-12-08

**Authors:** Shari R. Lipner, Mahmoud Ghannoum, Molly A. Hinshaw, Phoebe Rich, Beth S. Ruben, Tracey C. Vlahovic, Richard K. Scher

**Affiliations:** ^a^Department of Dermatology, Weill Cornell Medicine, New York, New York, USA; ^b^Center for Medical Mycology, Department of Dermatology, Case Western Reserve University and University Hospitals Cleveland Medical Center, Cleveland, Ohio, USA; ^c^Department of Dermatology, University of Wisconsin School of Medicine and Public Health, Madison, Wisconsin, USA; ^d^Department of Dermatology, Dermatology, Oregon Health and Science University, Portland, Oregon, USA; ^e^Department of Dermatology and Pathology, University of California, San Francisco, California, USA; ^f^Palo Alto Medical Foundation, Palo Alto, California, USA; ^g^Department of Podiatric Medicine, Temple University School of Podiatric Medicine, Philadelphia, Pennsylvania, USA

**Keywords:** SARS-CoV-2, COVID-19, Coronavirus, Pandemic, Onychomycosis, Fungal nail infection, Nail disease, Telemedicine, Nail clipping, Dermoscopy, Onychoscopy, Potassium hydroxide, Microscopy, Fungal culture, Polymerase chain reaction, Dermatology, Podiatry, Dermatopathology, Mycology

## Abstract

Onychomycosis is the most common nail condition seen in clinical practice, with significant impact on quality of life. Clinical examination alone is insufficient for accurate diagnosis, but mycological confirmation can be challenging during the COVID-19 pandemic. In this letter, a multidisciplinary panel of dermatologists, a podiatrist, dermatopathologists, and a mycologist, discuss considerations for mycological sampling during the pandemic.

Onychomycosis represents 50% of nail disorders seen in clinical practice, causing physical, social-emotional, and aesthetic consequences [1, 2]. Diagnosis relies on a thorough history and physical examination, aided by onychoscopy, and followed by mycological corroboration [3]. Confirmatory testing is necessary prior to initiating treatment to avoid incorrect diagnoses, treatment failures, drug-drug interactions, and adverse events; it is also cost-effective [4]. Until recently, in-person office visits were the norm for patients with nail problems. However, during the COVID-19 pandemic, telemedicine has been increasingly utilized to diagnosis and treat nail disorders [5], with patients requesting therapy without diagnosis confirmation. In this letter, a multidisciplinary panel of dermatologists, a podiatrist, dermatopathologists, and a mycologist, discuss considerations for mycological sampling during these unprecedented times.

For patients with clinical examination suggestive of onychomycosis, in-office physician sampling, patient-initiated nail specimens, or temporarily deferring diagnosis and treatment could be considered during the COVID-19 pandemic. Diagnostic options include potassium hydroxide with direct microscopy, fungal culture, and/or polymerase chain reaction (PCR) on subungual debris, or histopathology on nail plate clippings [2]. The ideal test ascertains identity and viability of the infecting organism, is inexpensive, rapid, and highly sensitive and specific. Direct examination has the highest sensitivity [6], is the least expensive technique ($6–$11) [7], and is the preferred screening method by this panel (Fig. 1). PCR and/or fungal culture can subsequently be used for fungal identification. If direct examination is positive and culture is negative, the culture could be repeated with different media. In cases with negative potassium hydroxide, where there is high clinical suspicion of onychomycosis, direct examination may be repeated or PCR or histopathology may be performed to increase sensitivity. Using direct examination, histopathology and fungal culture in combination have the highest positivity rate [6]. In-office onychoscopy is also inexpensive and helpful in distinguishing onychomycosis from other nail disorders in areas where mycology is not accessible [8].

We discourage patient-initiated sampling for diagnosis of onychomycosis. Mycological sampling requires specific knowledge of the pathogenesis of onychomycosis and specialized technical skills, which are refined during dermatology or podiatry residency. The specimen must be taken from the relevant part of the nail which differs for distal-lateral and proximal-subungual onychomycosis (Fig. 2, 3). For all types of testing with the exception of histopathology, the nail must be thoroughly cleaned to avoid contaminants (bacterial and/or nondermatophyte molds). In addition, a dual-action or heavy-duty nipper is necessary to obtain an adequately sized sample (>5 × 2 mm), as standard nail clippers are inferior [9, 10]. A #1 curette is preferred for obtaining subungual debris; slow careful technique and practice ensure painless sampling (Fig. 4; online suppl. Video 1; for all online suppl. material, see www.karger.com/doi/10.1159/000520628). In a study of 30 patients with nail findings suspicious for onychomycosis, samples were obtained from the nail plate, nail bed, and subungual debris by experienced podiatrists. Fungal cultures from subungual debris were >3 times more likely to be positive for dermatophytes than those from nail plate clippings [11]. In addition, there was higher concordance between office and central laboratory fungal cultures obtained by dermatologists and podiatrists compared to primary-care physicians, indicating that proper sampling technique translates into more accurate mycological testing [12]. Therefore, patient-obtained nail samples are likely to yield a high percentage of false-negative results with incorrect diagnoses and increasing diagnostic costs.

Therefore, we recommend that patients with suspected onychomycosis either schedule an in-person visit with a board-certified dermatologist or podiatrist, considering COVID-19 prevalence and patient risk factors, or defer diagnosis and treatment until an in-office visit is practical. Since the vast majority of onychomycosis cases are not life-threatening, and even with appropriate treatment clinical cure is limited by the slow physiologic nail plate growth rate, deferring treatment is a reasonable option. If there is accompanying interweb or plantar scale, empiric treatment of the skin with a low-cost topical antifungal could be considered.

Mycological confirmation of onychomycosis is necessary and cost-effective; these principles remain true even during the COVID-19 pandemic. We recommend against patient-initiated sampling. Physician expertise and training are required for proper mycological nail sampling, involving removal of maximum fungal load, which is part of the treatment. In regions of high COVID-19 prevalence or in patients at high risk for severe infection by the virus, diagnosis and treatment of onychomycosis should be postponed until nail sampling is safe and practical.

## Statement of Ethics

Subjects have given their written informed consent to publish their case including publication of images.

## Conflict of Interest Statement

Drs. Lipner, Ghannoum, Hinshaw, Rich, Ruben, Vlahovic, and Scher have no conflicts of interest relevant to the content of the submission.

## Funding Sources

No funding was received for this study.

## Author Contributions

Dr. Lipner contributed to conception, drafting, editing, and supervision. Drs. Ghannoum, Hinshaw, Rich, Ruben, Vlahovic, and Scher contributed equally, editing.

## Figures and Tables

**Fig. 1:**
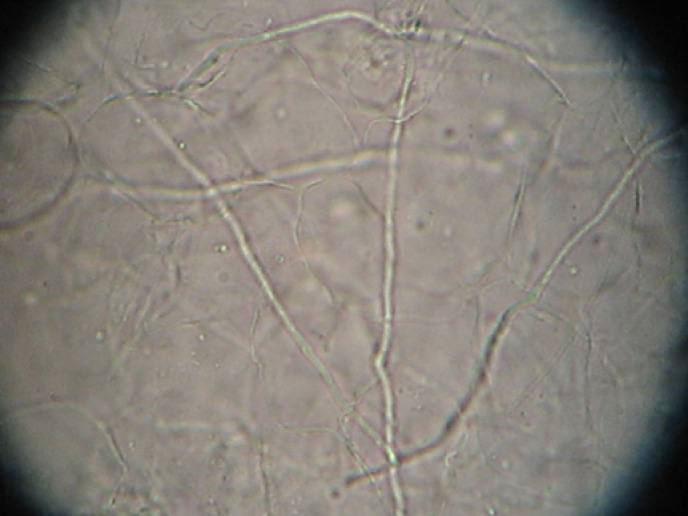
KOH staining on subungual debris. The long septate hyphae are dermatophytes, which are the most common fungi causing onychomycosis. Magnification ×400. KOH, potassium hydroxide.

**Fig. 2:**
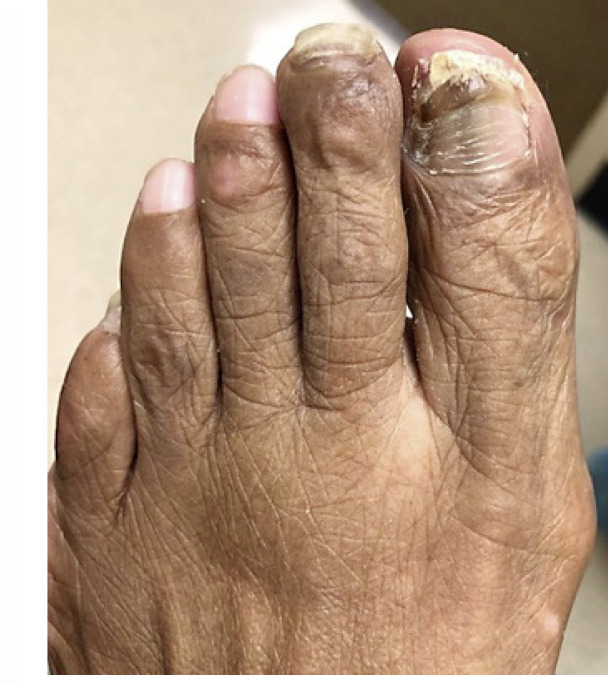
Right great and 2nd toenails before nail plate clipping.

**Fig. 3:**
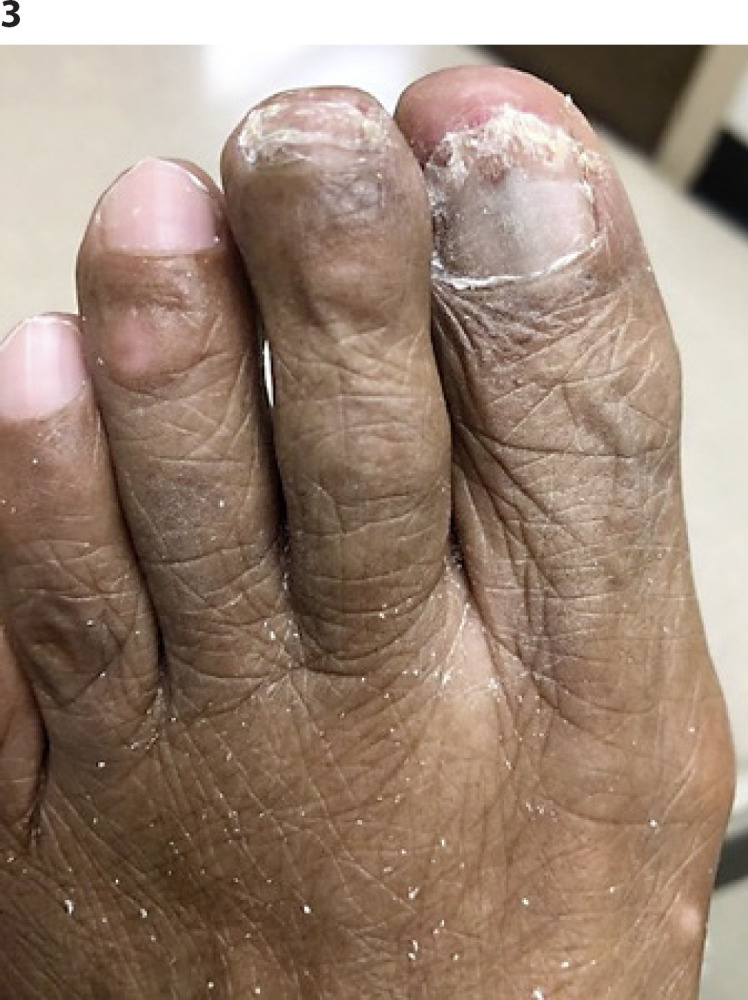
Patient from Figure 2, after nail plate clipping showing sampling of relevant area and removal of fungal load.

**Fig. 4:**
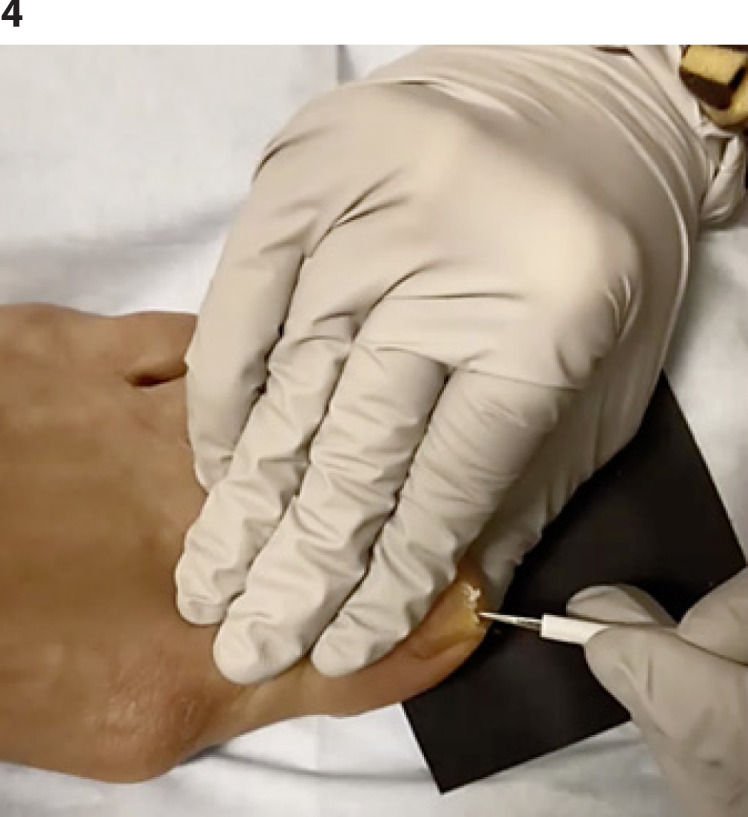
Mycological nail sampling. After cleaning the nail with soap and water and alcohol, the nail plate is clipped with a double-action nail nipper. A #1 curette is used to slowly and carefully scrape the subungual debris for analysis by KOH with direct microscopy, fungal culture, and/or PCR. Use of a black background provides contrast to ensure sufficient sample is collected (see also online suppl. Video 1). KOH, potassium hydroxide; PCR, polymerase chain reaction.
